# Capsaicin alleviates doxorubicin-induced acute myocardial injury by regulating iron homeostasis and PI3K-Akt signaling pathway

**DOI:** 10.18632/aging.205138

**Published:** 2023-11-01

**Authors:** Longbin Wang, Ying Liu, Si Li, Zhijian Zha, Yu Chen, Qi Wang, Shujing Zhou, Xufeng Huang, Ming Xu

**Affiliations:** 1 College of Clinical Veterinary Medicine, Huazhong Agricultural University, Wuhan, Hubei, China; 2 Department of Cardiology, Sixth Medical Center, PLA General Hospital, Beijing, China; 3 Department of Epidemiology and Biostatistics, School of Public Health, Tongji Medical College, Huazhong University of Science and Technology, Wuhan, Hubei, China; 4 Chinese Internal Medicine, Shanxi University of Chinese Medicine, Taiyuan, China; 5 School of Tropical Agriculture and Forestry, Hainan University, Haikou, China; 6 Department of Gastroenterology, Affiliated Hospital of Jiangsu University, Jiangsu University, Zhenjiang, China; 7 Faculty of Medicine, University of Debrecen, Debrecen, Hungary, China

**Keywords:** capsaicin, doxorubicin, ferroptosis, apoptosis

## Abstract

Background: Capsaicin (CAP), a frequently occurring alkaloid component found in spicy peppers, has demonstrated therapeutic potential against tumors, metabolic disease, and cardiovascular disorders. Doxorubicin (DOX), a widely used anthracycline drug in chemotherapy, is notorious for its cardiotoxicity. This study aimed to investigate the potential of CAP in mitigating DOX toxicity in mouse hearts and H9C2 cells, as well as to explore the underlying mechanisms.

Methods: In our study, we conducted experiments on both mice and H9C2 cells. The mice were divided into four groups and treated with different substances: normal saline, CAP, DOX and CAP+DOX. We evaluated the induction of ferroptosis by DOX and the remission of ferroptosis by CAP using various methods, including echocardiography, Hematoxylin and Eosin (H&E) staining, Masson’s trichrome staining, and determination of ferroptosis metabolites, genes and proteins. Additionally, we employed RNA-seq to identify the inhibitory effect of CAP on DOX-induced myocardial apoptosis, which was further confirmed through western blotting. Similar approaches were applied to H9C2 cells, yielding reliable results.

Results: Our study demonstrated that treatment with CAP improved the survival rate of DOX-treated mice and reduced myocardial injury. Mechanistically, CAP downregulated transferrin (Trf) and upregulated solute carrier family 40 member 1 (SLC40A1), which helped maintain iron levels in the cells and prevent ferroptosis. Furthermore, CAP inhibited DOX-induced apoptosis by modulating the phosphoinositide 3-kinase (PI3K)- protein kinase B (Akt) signaling pathway. Specifically, CAP activated the PI3K-Akt pathway and regulated downstream BCL2 and BAX to mitigate DOX-induced apoptosis. Therefore, our results suggest that CAP effectively alleviates acute myocardial injury induced by DOX.

Conclusion: Our findings demonstrate that CAP has the potential to alleviate DOX-induced ferroptosis by regulating iron homeostasis. Additionally, it can inhibit DOX-induced apoptosis by activating PI3K-Akt signaling pathway.

## INTRODUCTION

Doxorubicin (DOX), a member of the anthracycline family, is widely used in the treatment of various cancers [1]. However, its clinical use is limited due to its cardiotoxic effects. The acute cardiac events caused by DOX are primarily mediated through the regulation of ferroptosis, autophagy and apoptosis of cardiomyocytes [2]. In clinical practice, physicians determine the dosage based on the patient’s body surface area and administer anticardiotoxic drugs to reduce the cardiotoxic effects of DOX [3]. Dexrazoxane, an iron chelator that binds free iron, is currently the only cardiac protective drug approved by the US Food and Drug Administration for reducing the undesired side effects of DOX in advanced breast cancer [4, 5]. However, it is important to note that despite its efficacy in reducing the cardiotoxic effects of anthracyclines, dexrazoxane carries an increased risk of developing secondary malignant neoplasms and its use in pediatric patients is banned [6, 7]. Therefore, there is a need to explore new methods for mitigating DOX-induced myocardial injury, which holds clinical significance.

Ferroptosis is a recently discovered form of programmed cell death, characterized by iron-dependent cell death, accumulation of iron and lipid peroxidation [8]. It primarily occurs in tumors, neurodegenerative diseases, and anthracycline-induced cardiotoxicity [9]. Ferroptosis has emerged as a key player in the pathogenesis of DOX-induced cardiomyopathy, a severe side effect of the widely used chemotherapy drug DOX [10, 11]. Two important factors involved in ferroptosis are transferrin (TRF) and solute carrier family 40 member 1 (SLC40A1), also known as ferroportin. TRF, a glycoprotein, functions as an iron transporter in the bloodstream. It binds iron and facilitates its delivery to cells through interaction with the transferrin receptor. Conversely, SLC40A1 serves as an essential regulator for iron export from cells into the circulation. The balance between these two components has a profound influence on intracellular iron levels. Consequently, this balance also affects the susceptibility of cells to ferroptosis.

Apoptosis, a tightly regulated form of programmed cell death, also plays a crucial role in the development of DOX-induced cardiomyopathy [10]. Apoptosis is intimately connected with the phosphoinositide 3-kinase (PI3K)– protein kinase B (Akt or PKB) signaling pathway, commonly referred as the PI3K-Akt pathway [12]. This pathway plays a critical role in cell survival and proliferation. Akt, a key downstream effector of PI3K, phosphorylates a variety of substrates that govern apoptosis. It can directly inhibit pro-apoptotic factors like Bax and caspase-9, while activating anti-apoptotic proteins like BCL-2 [13–15].

Known for its pungent taste, CAP has demonstrated substantial therapeutic potential across various health-related domains. Recent investigations have unveiled its antioxidative, anti-inflammatory, and anticancer attributes [16–20]. Notably, CAP has been found to influence cellular iron metabolism, potentially impacting iron-dependent processes [21]. Specifically, CAP has ability of inhibition ferroptosis in normal tissue to protect it, it regulates ferroptosis through combination with iron, but it also could induce cancer ferroptosis through regulation SLC7A11-GPX4 signaling pathway [22, 23]. Another particular note is its influence on the pivotal PI3K-Akt pathway. Studies have unveiled that CAP can modulate the PI3K-Akt pathway at multiple tiers [21, 24]. It can stimulate PI3K activity, initiating the phosphorylation and activation of Akt, which subsequently orchestrates downstream events associated with cell proliferation and survival. Therefore, further investigations into the roles of CAP, the PI3K-Akt pathway, and iron metabolism in disease are necessary.

Drawing upon previous research findings, we hypothesized that CAP may provide a protective effect against DOX-induced cardiac ferroptosis. Our experimental validation supported this hypothesis. Additionally, our investigations demonstrated that CAP could inhibit DOX-induced myocardial apoptosis, as revealed by RNA-seq analysis. This study successfully identified and characterized the specific pathways involved in these two types of cell death during the experimental procedures.

## RESULTS

### CAP alleviated DOX-induced myocardial injury

**Figure 1:**
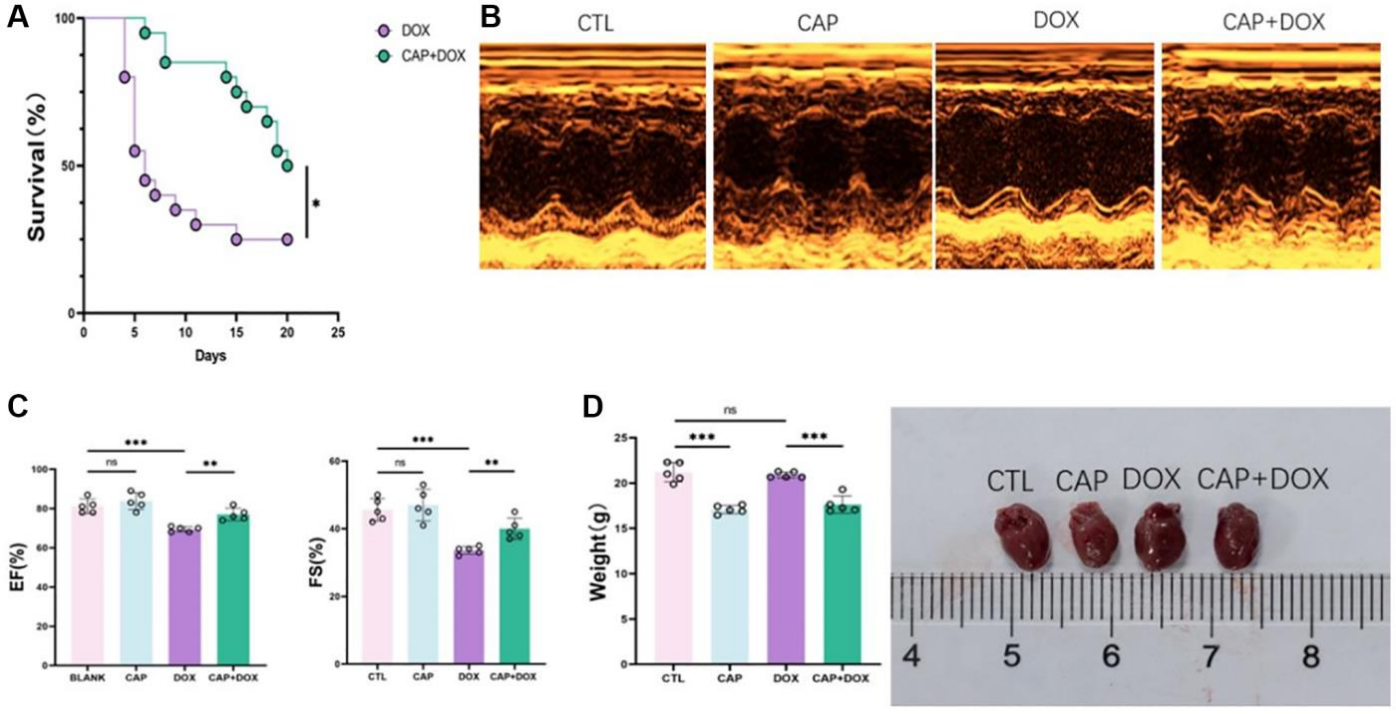
**CAP alleviated DOX-induced myocardial injury.** (**A**) Survival rate of mice injected with DOX or CAP+DOX (*n* = 20). (**B**) Echocardiographic images of CTL group, CAP group, DOX and DOX + CAP groups in at day 4 after injection. (**C**) Shortening fraction (left) and ejection fraction of mice at day 4 after injection (right) (*n* = 6). (**D**) Weight of mice (left) and anatomical appearance of mice heart (right) (*n* = 5). Data are shown as the mean ± SEM. Statistical significance was determined using two-tailed student’s *t*-test. ^*^p < 0.05, ^**^p < 0.01, ^***^p < 0.001.

**Figure 2:**
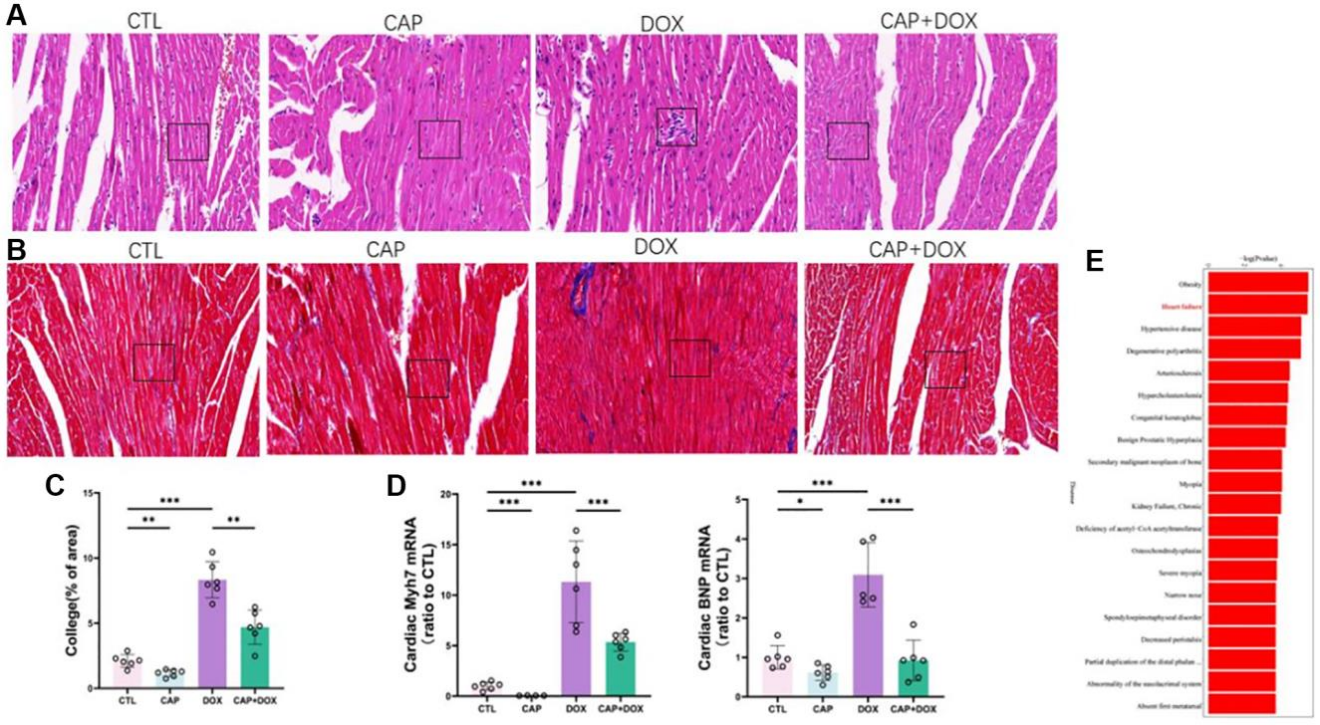
**CAP alleviated DOX-induced myocardial injury.** (**A**) Cross-sectional staining of mouse heart, HE staining. (**B**) Masson staining of Cross-sectional staining of mouse heart. Scale: 50 µM (**C**) Percentage analysis of collagen fibers (*n* = 6). (**D**) mRNA expression levels ofMYH7 (left) and BNP (right) in mouse heart tissue on day 4 after injection (*n* = 6). (**E**) KEGG disease enrichment map of mice in DOX and CAP+DOX groups (*n* = 3). Data are shown as the mean ± SEM. Statistical significance was determined using two-tailed student’s *t*-test. ^*^p < 0.05, ^**^p < 0.01, ^***^p < 0.001.

The survival rate of mice pretreated with CAP (Figure 1A) was higher than that of the DOX group during the observed 20 days. To investigate the gross changes in the mouse heart, we performed echocardiography on four groups of mice (Figure 1B) and found that DOX worsened HR FS EF in mice, but CAP prevented the deterioration of the hearts of mice in the DOX group, resulting in a corresponding improvement in the short-axis shortening ratio of the left ventricular (LV) (Figure 1C left) and the Ejection Fraction (EF) (Figure 1C right). Upon dissecting the mouse hearts, we found that the hearts of mice in the DOX group were larger than those in the control(CTL) group, while the hearts of mice in the CAP+DOX group were smaller than those in the DOX group (Figure 1D left), and when considering the mice’s weight (Figure 1D right), the compensatory increase in heart volume caused by the CAP+DOX group was reduced, thereby reducing the compensation of the heart due to the decreased blood supply caused by DOX, which was sufficient to support the weight of the mice. Tissue analysis of the mouse hearts using HE staining showed that the arrangement of myocardial cells in the DOX group was more disorderly than that in the other three groups, and mice in the CAP+DOX group was apparently better than those in the DOX group (Figure 2A). Using Masson staining to observe the content of collagen fibers (Figure 2B), the hearts of mice in the CAP+DOX group contained significantly less collagen fibers than those in the DOX group (Figure 2C). Measurement of heart failure indicators showed that BNP and MYH7 in the heart muscle of mice in the CAP+DOX group were notably lower than those in the DOX group (Figure 2D). The decrease in these two genes, which serve as indicators of heart failure, suggests that CAP is effective in reducing DOX-induced heart failure [25, 26]. Additionally, we found that the group treated with CAP alone also demonstrated better performance than the CTL group, and we believe that CAP did play a key role in protecting cardiovascular function, as previously reported in the literature. KEGG disease enrichment was used to analyze the CAP+DOX group and DOX group using RNA-seq methodology, revealing differences in disease between the CAP+DOX and DOX groups, with heart failure ranking second (Figure 2E). In conclusion, CAP not only protected the heart under normal circumstances but also protected against DOX-induced myocardial disease.

### CAP mitigated DOX-induced ferroptosis in mice

**Figure 3:**
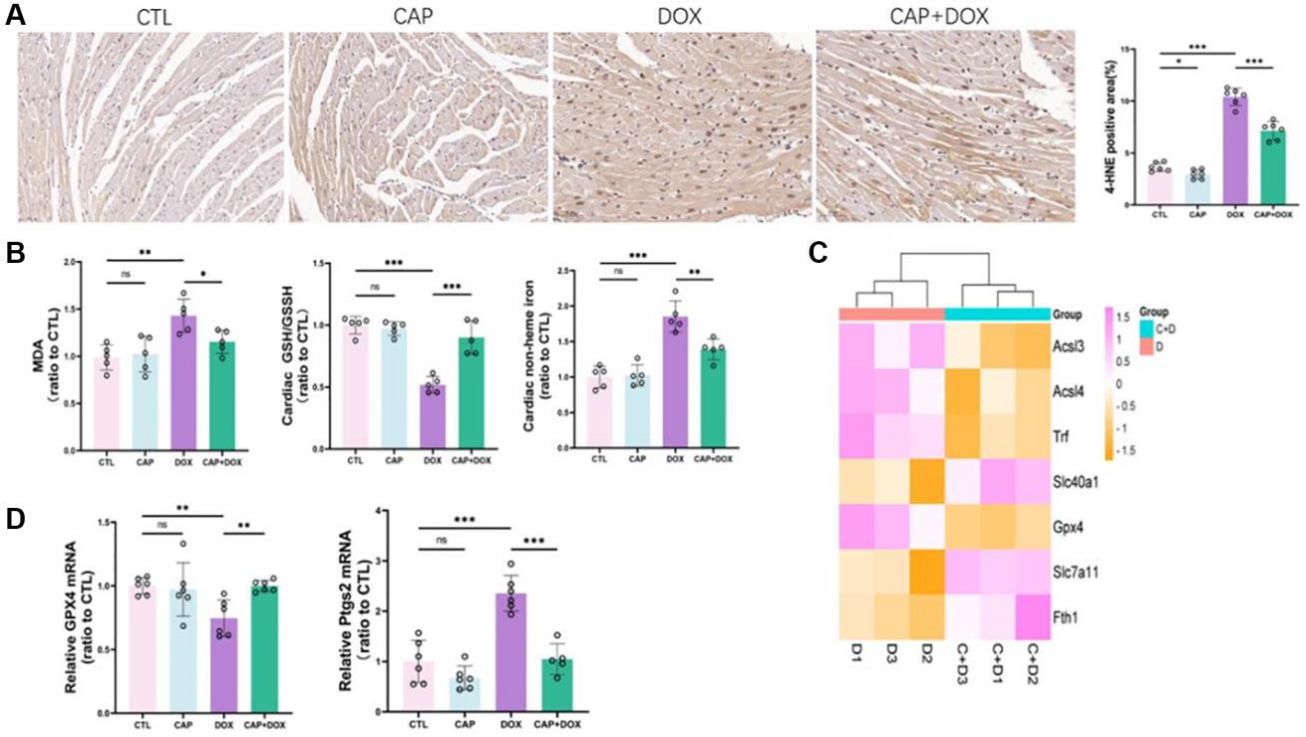
**CAP mitigated DOX-induced ferroptosis in mice.** (**A**) The cardiac tissues of mice from each group were stained with anti - 4-HNE (left) and quantitative analysis was performed (right). Scale: 50 µM (**B**) Mouse myocardial MDA (left), myocardial glutathione to reduced glutathione ratio (middle), myocardial iron non-heme iron (right) (*n* − 5). (**C**) Cluster heat maps of genes associated with ferroptosis in dox and CAP+DOX groups (*n* = 3). (**D**) mRNA expression levels of GPX4 (left) and PTGS2 (right) in mouse myocardium (*n* = 6). Data are shown as the mean ± SEM. Statistical significance was determined using two-tailed student’s *t*-test. ^*^p < 0.05, ^**^p < 0.01, ^***^p < 0.001.

**Figure 4:**
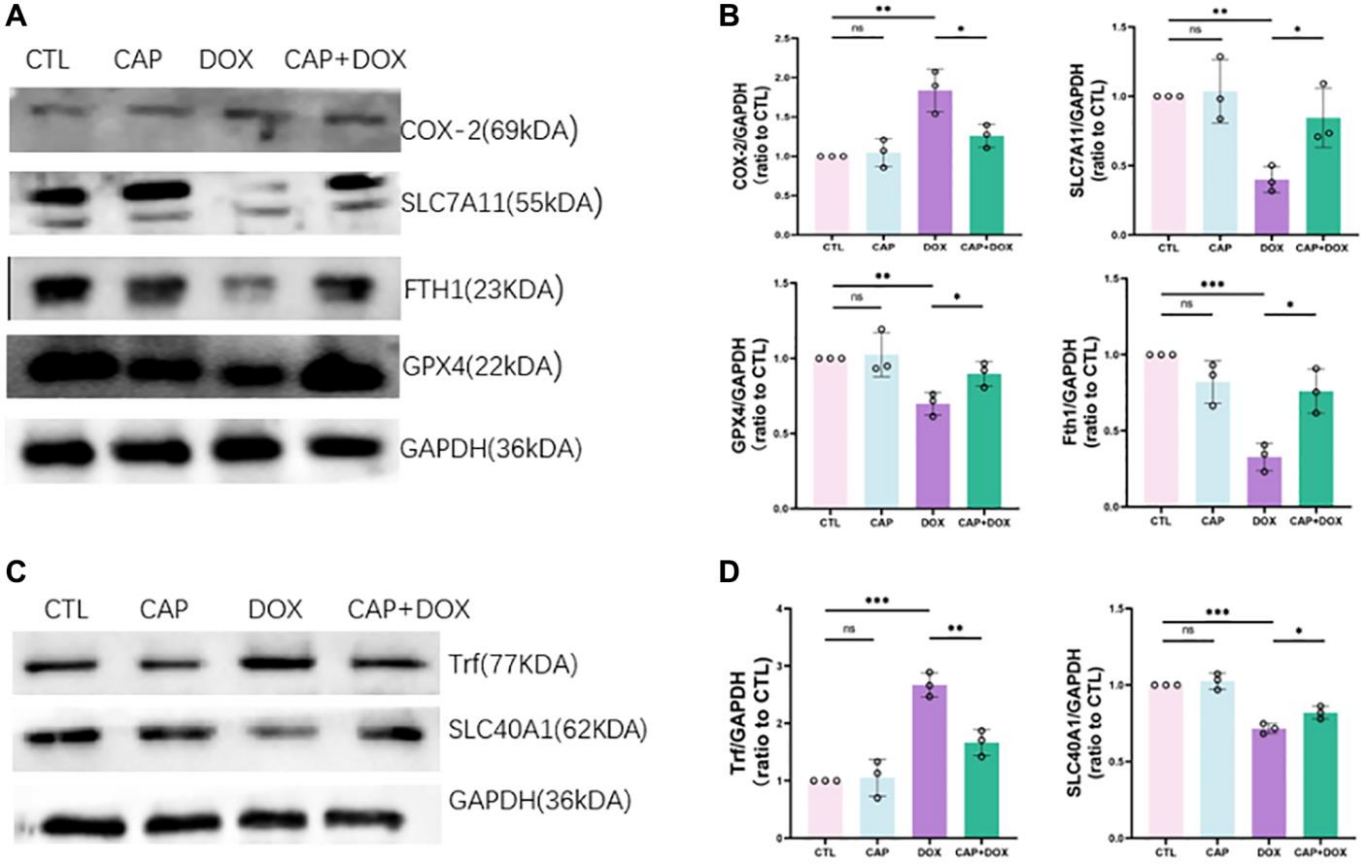
**CAP mitigated DOX-induced ferroptosis in mice.** (**A**) Immunoprotein gel of COX-2, SLC7All, FTHI, GPX4 in mice (*n* = 3). (**B**) Relative expressions of COX-2, SLC7All, FTHI, GPX4 in mice. (**C**) Immunoprotein gel of Trf and SLC40A1 in mice (*n* = 3). (**D**) Relative expression levels of Trf and SLC40A1 in mice (*n* = 3). Data are shown as the mean ± SEM. Statistical significance was determined using two-tailed student’s *t*-test. ^*^p < 0.05, ^**^p < 0.01, ^***^p < 0.001.

Ferroptosis has been reported to play an important role in doxorubicin-induced cardiomyopathy (DIC) [27, 28]. Our research showed that CAP inhibited DOX-induced cardiac ferroptosis. Firstly, the content of 4-HNE in the heart muscle of mice in the four groups was determined by immunohistochemistry, and it was found that the content of 4-HNE in the heart tissue of mice in the CAP+DOX group was apparently lower than that in the DOX group (Figure 3A). As a product of lipid peroxidation, the decreased level of 4-HNE indicated that CAP reduced lipid peroxidation in cardiomyocytes. To further demonstrate that lipid peroxidation was inhibited, MDA, another product of this process, was detected, and as expected, MDA content in CAP+DOX group was also significantly lower than that in DOX group (Figure 3B, left). Furthermore, our results provide evidence that CAP reduces lipid peroxidation induced by DOX. In order to further confirm that CAP inhibits DOX-induced ferroptosis, and that accumulation of iron ions and depletion of GSH are key to ferroptosis, we detected non-heme iron content and GSH/GSSH content in mouse myocardium (Figure 3B, middle), and found that CAP did inhibit the occurrence of DOX-induced ferroptosis. Meanwhile, we also found that the cardiac iron in CAP+DOX group lower than DOX (Figure 3B, right). We found through RNA-seq that CAP had a important effect on DOX-induced ferroptosis, and we expressed the genes with notably differences related to ferroptosis using cluster heat maps (Figure 3C). Later, we verified the related genes in the figure. Firstly, mRNA of GPX4, the core factor of ferroptosis, and PTGS2, the product of ferroptosis, were performed. CAP could indeed apparently inhibit DOX-induced ferroptosis (Figure 3D, left). GPX4, as the core factor of ferroptosis, was significantly up-regulated in CAP+DOX (Figure 3D, right), and PTGS2, as a result of ferroptosis, was also decreased. We then performed protein-related verification and found that changes in GPX4 and PTGS2 were consistent with mRNA, and SLC7A11 upstream of GPX4 was also elevated (Figure 4A, 4B). In addition, FTH1, as an important protein for the treatment of ferrous ions, was also up-regulated, and the expression of FTH1 in CAP+DOX group was significantly higher than that in DOX group (Figure 4A, 4B).

Through RNA-seq analysis, we have discovered that CAP regulated two key components of intracellular iron content, SLC40A1 and Transferrin (TRF). SLC40A1, also known as FPN1, encodes the iron transport protein that exports iron out of cells, while TRF allows iron to be taken up by cells by binding to transferrin receptors (TFR) [29, 30]. Our experimental results have shown that CAP is capable of upregulating or downregulating SLC40A1 and TRF, thereby regulating intracellular iron content and inhibiting DOX-induced ferroptosis (Figure 4C, 4D). These findings indicated that CAP modulated ferroptosis by adjusting intracellular iron content.

### CAP inhibited DOX-induced ferroptosis in H9C2 cells

**Figure 5:**
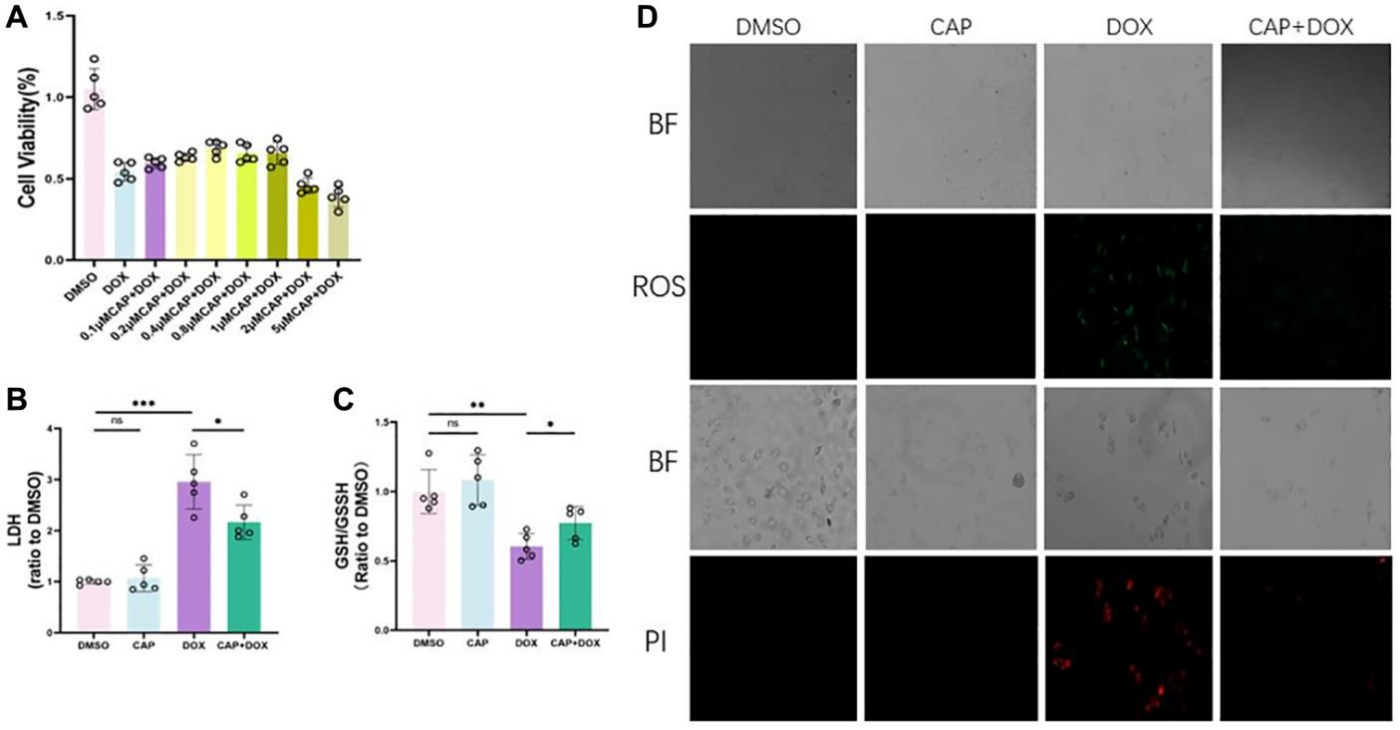
**CAP inhibited DOX-induced ferroptosis in H9C2 cells.** (**A**) H9C2 cells were treated with 2.5 µM DMSO or DOX or varying concentrations of CAP+ 2.5 µM DOX for 6 hours, followed by measurement of cell viability using cck-8 assay (*n* = 5). (**B**) H9C2 cells were treated with 2.5 µM DMSO or DOX or 0.4 µM CAP+ 2.5 µM DOX for 6 hours, followed by measurement of cell toxicity using LOH assay (*n* = 5). (**C**) H9C2 cells were treated as described previously for 6 hours, followed by measurement of intracellular GSH/GSSG levels (*n* = 5). (**D**) H9C2 cells were treated with 2.5 µM DMSO or DOX or 0.4 µM CAP + 2.5 µM DOX for 24 hours, and then observed under bright field (BF) and fluorescence modes for measurement of intracellular ROS levels (top) and cell death(bottom) (*n* = 3). Scale: 20 µM Data are shown as the mean ± SEM. Statistical significance was determined using two-tailed student’s *t*-test. ^*^p < 0.05, ^**^p < 0.01, ^***^p < 0001.

**Figure 6:**
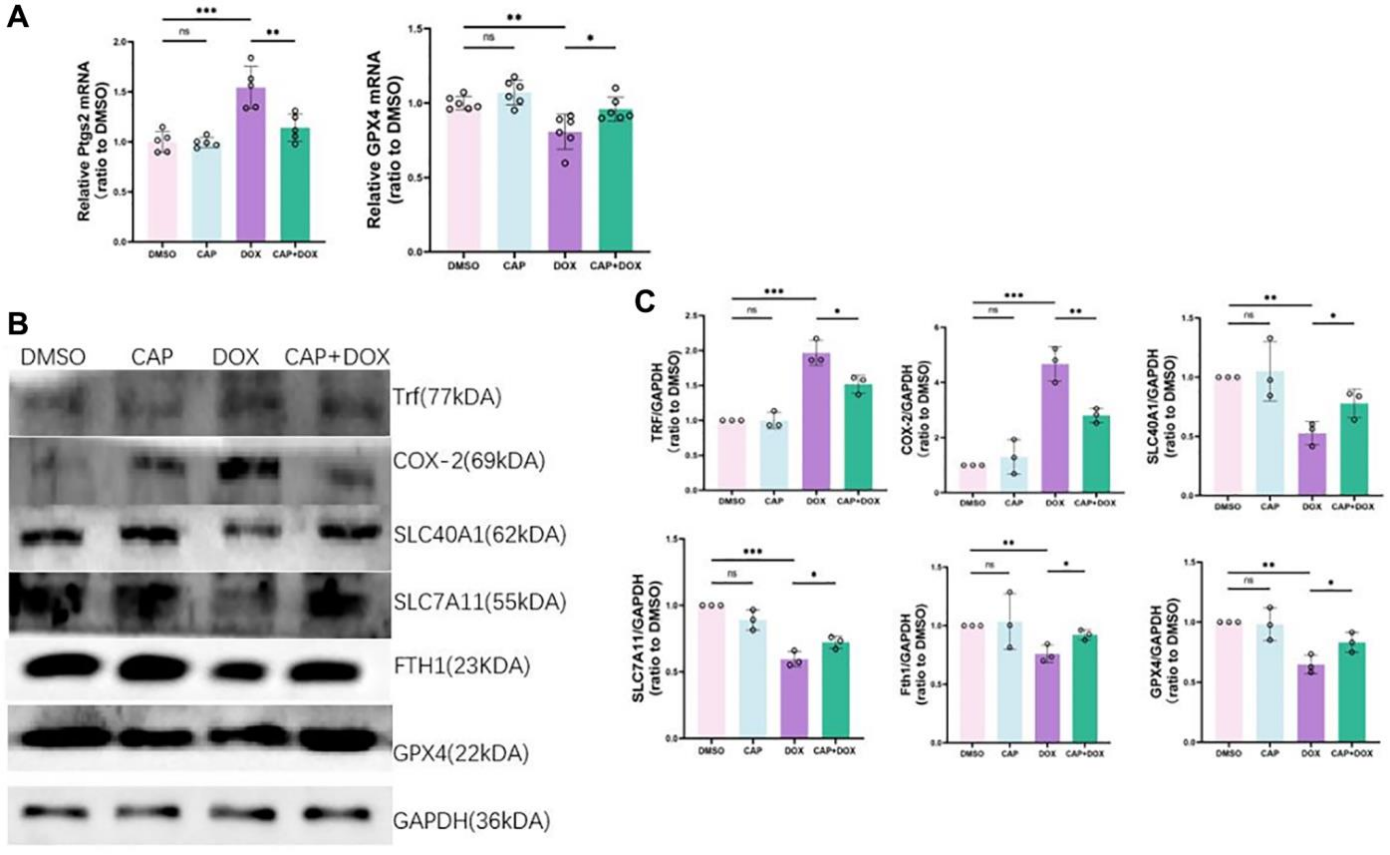
**CAP inhibited DOX-induced ferroptosis in H9C2 cells.** (**A**) H9C2 cells were treated with 2.5 µM DMSO or DOX or 0.4 µM CAP+ 2.5 µM DOX for 6 hours, and then, qPCR was used for quantitative analysis of PTGS2 (right) and GPX4 (left) mRNA expression (*n* = 5). (**B**) Relative expressions of COX-2, SLC7All, FTHl, GPX4, TRF, SLC40Al in H9C2 (*n* = 3). (**C**) H9C2 cells were treated with 2.5 µM DMSO or DOX or 0.4 µM CAP+ 2.5 µM DOX for 8 or 12 hours, followed by extraction of proteins for Western blot analysis (*n* = 3). Data are shown as the mean ± SEM. Statistical significance was determined using two-tailed student’s *t*-test. ^*^p < 0.05, ^**^p < 0.01, ^***^p < 0.001.

To demonstrate that CAP exerted the same effect *in vitro*, we performed an *in vitro* experiment using H9C2 cells [31]. Methods described in previous literature were used to treat H9C2 with DOX and CAP. Firstly, we conducted a CCK-8 cell viability assay and found that CAP significantly increased cell viability in the DOX group (Figure 5A). We then performed a lactate dehydrogenase (LDH) cytotoxicity assay (Figure 5B) and found that CAP also reduced DOX-induced cell toxicity. In order to detect the occurrence of ferroptosis, we first measured the ratio of reduced glutathione to oxidized glutathione in cells, and found that CAP up-regulated this ratio (Figure 5C). We then tested for intracellular reactive oxygen species and cell death, and found that the CAP+DOX group was lower than the DOX group as evidenced by ROS and PI staining (Figure 5D). Finally, we measured the mRNA and protein levels of ferroptosis-related genes. The CAP+DOX group was elevated in the expression of GPX4 mRNA compared to the DOX group, while PTGS2, which is induced by DOX, was significantly reduced by CAP (Figure 6A). Testing for GPX4, COX-2, and FTH1 protein levels revealed that GPX4 and FTH1 were higher in the CAP+DOX group than in the DOX group, whereas COX-2 decreased in the CAP+DOX group (Figure 6B, 6C). Meanwhile, we validated the effects of TRF and SLC40A1 on cellular iron intake by modulating their expression levels *in vitro* (Figure 6B, 6C). Our results indicate that CAP regulated intracellular iron pool, iron metabolism to inhibit dox-induced ferroptosis by up- or down-regulating the expression of TRF and SLC40A1.

### CAP relieved DOX-induced apoptosis of cardiomyocytes by regulating PI3K-Akt signaling pathway

**Figure 7:**
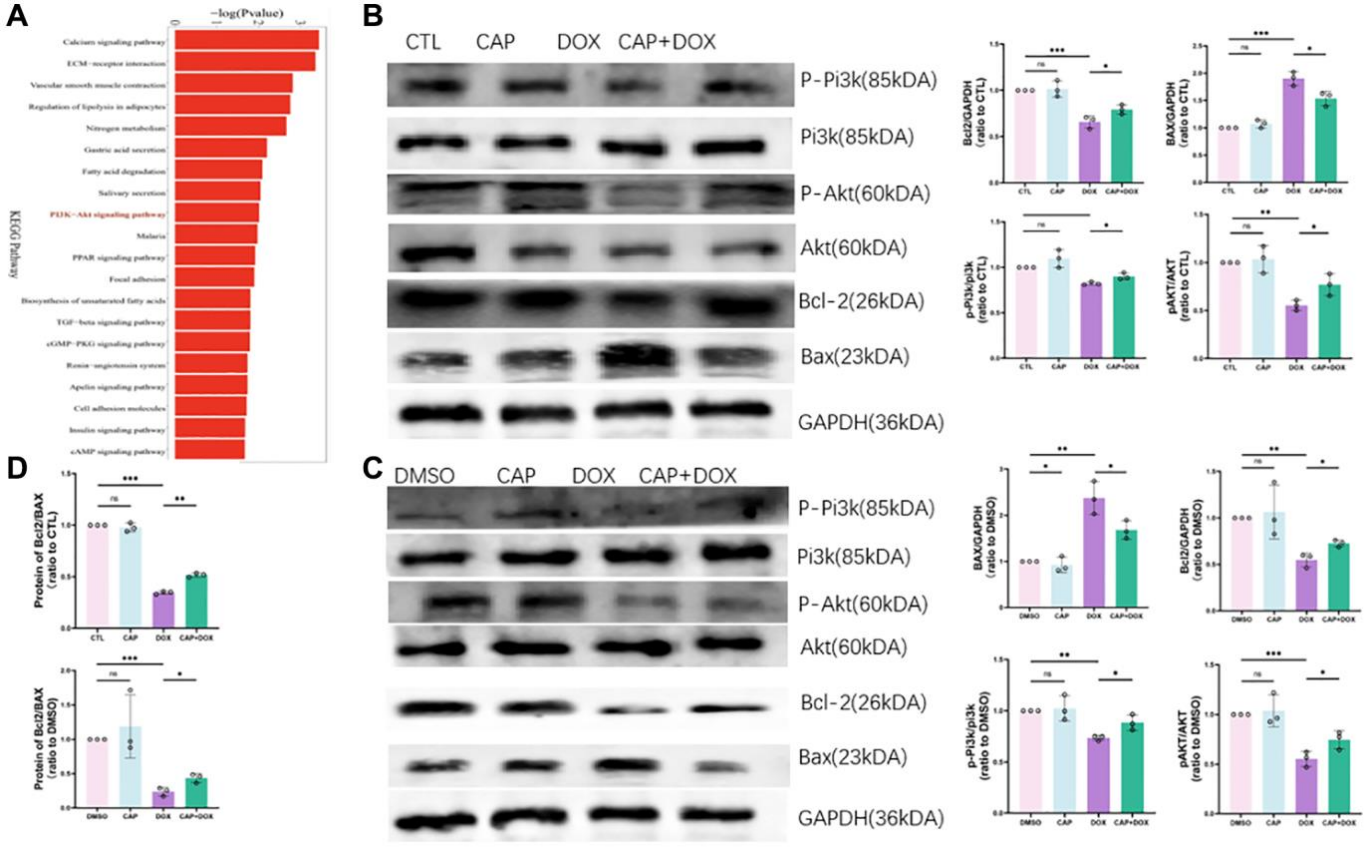
**CAP relieved DOX-induced apoptosis of cardiomyocytes by regulating PI3K-Akt signaling pathway.** (**A**) KEGG pathway enrichment plots for DOX group and CAP+DOX group 24 hours after injection of DOX (*n* = 3). (**B**) Quantitative analysis of heart tissue collected from four groups of mice after injection of saline or CAP or DOX or CAP pretreatment followed by DOX treatment, using immunoblotting (*n* = 3). (**C**) Quantitative and analytical immunoblotting for H9C2 cells treated with 2.5 µM DMSO, DOX or 0.4 µM CAP+2.5 µM DOX for 12 hours (*n* = 3). (**D**) Ratio of the BCL2 and BAX proteins in mouse myocardial tissue (top) and in H9C2 cells (bottom), used to indicate risk of apoptosis (*n* = 3). Data are shown as the mean ± SEM. Statistical significance was determined using two-tailed student’s *t*-test. ^*^p < 0.05, ^**^p < 0.01, ^***^p < 0.001.

As mentioned earlier, ferroptosis plays an important role in DOX-induced myocardial disease; however, apoptosis, as a common form of cell death, also plays a key role in DOX-induced myocardial disease [29]. DOX induced cardiomyocyte apoptosis by suppressing the PI3K-Akt pathway and regulating BCL2 and BAX [10]. Through KEGG pathway enrichment analysis, we discovered that the calcium ion channel and PI3K-Akt pathway were significantly enriched (Figure 7A). Therefore, to investigate the effect of CAP on DOX-induced apoptosis, we measured the mRNA and protein levels of apoptosis-related factors and found that BCL2, which was inhibited by DOX, was elevated in the presence of CAP, while DOX-induced BAX was decreased (Figure 7B). The ratio of BCL2 to BAX could be used to determine the degree of myocardial apoptosis [32]; we found that CAP inhibited DOX-induced apoptosis (Figure 7D top). We then validated the phosphorylation levels of PI3K and Akt upstream and found that DOX reduced the phosphorylation levels of PI3K and Akt, whereas CAP+DOX restored the phosphorylation levels of PI3K and Akt. Therefore, we concluded that CAP alleviated DOX-induced apoptosis by upregulating the phosphorylation levels of PI3K and Akt. CAP inhibited DOX-induced myocardial apoptosis in mice, so we replicated similar experiments at the cellular level. Through our experiments, we found that CAP suppressed DOX-induced apoptosis in H9C2 cells. We also performed PI3K-Akt pathway validation in our cellular experiments and obtained similar results (Figure 7C). We then analyzed the relevant apoptosis-related proteins and found that BCL2, as an anti-apoptotic protein [33], was significantly upregulated by CAP when DOX inhibits this gene; on the other hand, BAX, as a pro-apoptotic protein [34], was inhibited by CAP (Figure 7C). And the ratio of BCL2 and BAX decreased, so we could tell the apoptosis was reduced by CAP (Figure 7D bottom). We have determined that CAP activated the PI3K-Akt pathway and reduced DOX-induced cellular apoptosis.

## DISCUSSION

CAP, a common substance found in chili peppers, is easily ingested through diet [35]. This study confirms that CAP reduced the cardiac toxicity of anthracycline drugs, which has important clinical implications for patients undergoing chemotherapy with anthracyclines. In our experiments, we found that CAP regulated intracellular iron pools to improve iron-induced cell death. Through RNA-seq, we also observed significant differences in members of the acyl-CoA synthetase long-chain (ACSL) family, including ACSL1, ACSL2, ACSL3, ACSL4, and ACSL6, between the CAP+DOX group and the DOX group. The ACSL family activates fatty acids by conjugating them with CoA group and providing substrates for specific metabolic pathways. ASCL4 and ACSL3 are critical for iron-induced cell death mediated by arachidonic acid/adrenaline and linoleic acid, respectively. ACSL4 helps cells synthesize PUFAs, which promotes ferroptosis [36]. Several studies have shown that inhibiting the expression of ACSL4 inhibited the occurrence of iron-induced cell death [36–38]. ACSL3 activates monounsaturated fatty acids (MUFA) to competitively inhibit PUFA-induced ferroptosis [39]. Interestingly, we conducted mRNA quantification on ACSL4 and ACSL3, and in our experiments, we found that CAP decreased the expression of ACSL4 mRNA induced by DOX. However, contrary to our expectations, ACSL4 did not increase in the DOX group compared to the CTL group, but decreased instead. We speculate that this may be due to different pathways of iron-induced death in DOX and ACSL4, and negative feedback mechanisms resulted in lower expression of ACSL4 in the DOX group compared to the CTL group.

Additionally, during our survival rate tests on mice, we found that CAP notably improved initial survival in acute myocardial toxicity, prolonging the survival time of mice, but after 10 days, the relative mortality rate of CAP mice was higher than that of the DOX group. We hypothesized this might be due to the higher metabolic rate of CAP compared to DOX, resulting in early metabolism of CAP before it was completely metabolized, while DOX was not entirely metabolized [40, 41]. However, further drug metabolism analysis is necessary to determine the specific situation. Furthermore, we also found that high concentrations of CAP had damaging effects on mice and H9C2, promoting apoptosis. We suspected that the dose of CAP was crucial in this treatment process, which is concerned with releasing neuropeptides [42]. In this study, we found the most appropriate dose through the drug concentration gradient experiment, but in future studies, the dose will need to be more precisely calibrated to achieve better therapeutic effects.

When comparing the CAP and CAP+DOX groups of mice individually, we found that CAP had a protective effect on the myocardium, as indicated by improved levels of BNP and MYH7 in the CAP group compared to the CTL group.

SLC40A1, TRF, and FTH1/FTL binding proteins are important proteins for regulating intracellular iron content, playing a critical role in the regulation of ferroptosis. Iron outflow mediated by SLC40A1 and iron protein output mediated by exosomes suppressed ferroptosis. TRF bond with Fe^3+^ to form holo-TF, which then binds with transferrin receptor and enters the nucleus through endocytosis, thereby increasing the intracellular iron content [43]. CAP up-regulated the expression of SLC40A1 and at the same time inhibited the uptake of iron by Trf, leading to a decrease in intracellular iron content [44]. Since the increase in intracellular iron content put the cell in a state prone to ferroptosis, CAP reduced the risk of ferroptosis by reducing iron content. The transcription of these genes was directly regulated by RNA polymerase, so we believed that CAP activated RNA polymerase through some pathway, and the specific pathway needed to be verified by further experiments.

PI3K-Akt is a very common pathway that involves a series of programmed cell deaths such as apoptosis, ferroptosis, and autophagy [12, 45–46]. This study found that CAP up-regulated PI3K-Akt to inhibit ferroptosis. However, there were other patterns of cell death that are affected by this pathway, so it needs to be verified whether CAP regulates other cell death pathways such as autophagy and ferroptosis through this pathway. In addition, the upstream pathway of PI3K-Akt was very complex, and we speculated that CAP might induce changes in intracellular calcium ion levels through activation of TRPV1, thereby affecting this pathway [47]. However, since our experiment focused on PI3K-Akt, the specific conduction of upstream signals needs to be further verified.

Regarding the clinical significance of CAP in alleviating DOX-induced myocardial ferroptosis, Physicians could determine a patient’s dietary history before administering anthracycline drugs. But people also might take CAP in their daily diet, which would cause physicians be difficult to calculate the amount of CAP to be used, so we suggested that before using CAP combined with DOX treatment, physicians need to wait for the completion of the metabolism of the original CAP in the patient’s body, and then use CAP combined with DOX treatment. Combining previous reports that DOX reduced the resistance of cancer cells to anti-cancer drug treatment, combination chemotherapy with CAP and anthracycline drugs theoretically appeared feasible, but further research and clinical data would be needed to support this, and treatment needs to be combined with the patient’s weight to adjust the dose of CAP, as this study was designed to explore the mechanism, it was limited. Therefore, we considered that the future clinical treatment of CAP still needed specific pharmacological research.

## CONCLUSION

CAP reduced DOX-induced myocardial ferroptosis by downregulating cellular iron levels and protect myocardial cells against DOX-induced apoptosis. Specifically, CAP inhibited DOX-induced myocardial ferroptosis by decreasing the expression of Trf and up-regulating the expression of SLC40A1. And CAP mitigated DOX-induced myocardial apoptosis by activating of PI3K-Akt signaling pathway. This study represents the first case to report the corresponding results.

## MATERIALS AND METHODS

### Mice and treatment

All protocols were approved by the Institutional Animal Care and Use Committee of the Huazhong Agricultural University (HZAUMO-2023-0128). Male C57BL/6J mice were housed in a temperature- and humidity-controlled room with normal illumination, fed a commercial diet (Animal Center of Huazhong Agricultural University), and given free access to water. Adult (6 to 8 weeks old) male animals were used in this study. The mice in capsaicin (CAP) + doxorubicin (DOX) group were subjected to one time intraperitoneal injection of CAP (HY-10448, MedchemExpress) in every 24 h for 3 days. The mice in CAP group were subjected to a single intraperitoneal injection of saline. The mice in DOX group were subjected to a single intraperitoneal injection of DOX (HY-15142, MedchemExpress, 20 mg/kg, body weight) for 1 or 4 days. The mice in CTL group were subjected to intraperitoneal injection of saline for the same time.

### Cell culture

H9C2 cells were cultured in Dulbecco’s modified Eagle’s medium (DMEM) (Gibco) containing 10% fetal bovine serum (FBS) and 1% antibiotics penicillin and streptomycin (P/S) at 37°C and 5% CO2 in the incubator. Cells were treated with DOX or DMSO with or without CAP, and the concentration and cells were determined according to the specific experimental requirements.

### Histopathological evaluation

Hearts of mice were fixed in 4% paraformaldehyde (pH 7.4) for several hours, embedded in paraffin, and serially sectioned at 5-µM thickness. The sections were stained with Hematoxylin and Eosin (H&E) for routine histological examination with a light microscope. To measure collagen deposits, select sections were stained with Masson. For each mouse, three adjacent sections were quantified using ImageJ software.

### Immunofluorescence and immunohistochemistry

Tissues were treated as previously described. Anti-4-hydroxynonenal antibody (ab216880, Bioss) was used to detect protein expression in the heart tissue. Images were captured at 50× with an Olympus immunofluorescence microscope. Quantification of the relative intensity of protein staining was performed by automated image analysis in five randomly chosen 200× fields for each sample.

### RT-qPCR

Total RNA was isolated from tissues or cells using Trizol (Vazyme), and RNA concentration and purity were measured using a spectrophotometry. RNA was reverse-transcribed using the PrimeScript RT reagent Kit (Vazyme) in accordance with the manufacturer’s instructions. Quantitative PCR was performed using lightcycle96 Real-Time System (Roche) in accordance with the manufacturer’s instructions. The fold difference in gene expression was calculated using the 2^−ΔΔCt^ method and is presented relative to GAPDH mRNA. All reactions were performed in triplicate. The primer sequences are in Supplementary Table 1.

### RNA sequencing and data analysis

Whole-genome gene expression analysis was performed using the heart tissues from DOX- and DOX+CAP-treated mice (*n* = 3 per group) at 24 h. The total RNA was extracted using Trizol (Vazyme), and cDNA samples were sequenced using a sequencing system (illumina novaseq 6000). The reference Mus musculus genome and gene information were downloaded from the National Center for Biotechnology Information database. Raw reads were filtered to produce high-quality clean data. All the subsequent analyses were performed with the clean data. All the differentially expressed genes were used for heat map analysis and KEGG ontology enrichment analyses. For KEGG enrichment analysis, a *P*-value < 0.05 was used as the threshold to determine significant enrichment of the gene sets.

### Biochemical analysis

Serum and cardiac malondialdehyde (MDA) levels were measured using a kit (S0131, Beyotime) in accordance with the manufacturer’s instructions. GSH/GSSG ratio was measured using a glutathione assay kit (No. S0052, Beyotime) according to the product instructions. The LDH kit was bought from Nanjing Jiancheng and was used to detect cytotoxicity according to the instructions.

### Cell viability assay

Cell viability assay was performed using the CellTiter-Glo^®^ Luminescent CCK-8 Cell Counting Kit (A311, Vazyme). Cells were seeded at 5 × 10^3^ cells per well in a 96-well opaque plate. After the indicated treatment, the luminescent signal was measured using GloMax-Multi Detection System (Promega) in accordance with the manufacturer’s instructions. Based on the recorded luminescence, the percentage of cell viability was then calculated accordingly.

### Measurement of ROS

H9C2 cells were seeded in a 24-well plate. Then the intracellular reactive oxygen species content was detected using Beyotime Reactive Oxygen kit (S0033S, Beyotime) according to the instructions.

### Cell death measurement

H9C2 cells were seeded in a 24-well plate. After the treatment, cell death was determined by propidium iodide (PI) staining (10 ng/mL) coupled with microscopy.

### Western blot analysis

Total proteins were extracted from the tissues by homogenizing in RIPA buffer containing protease inhibitors. The homogenate was cleared by centrifugation at 4°C for 30 min at 12,000 rpm, and the protein was collected. Protein concentration was measured using the BCA Protein Assay Kit (Vazyme). A total of 20 or 30 mg protein per sample was resolved in a 10–12% SDS-PAGE and transferred to a nitrocellulose membrane. The membranes were blocked with 5% skim milk in Tris-buffered saline containing 0.2% Tween-20, then incubated with primary antibodies at 4°C overnight. The following antibodies were used: anti-COX-2 (A1253, Abclonal), anti-SLC7A11 (CSB-PA145393, CUSABIO) anti-FTH1 (WL05360, Wanleibio), anti-GPX4 (A11243, Abclonal), anti-GAPDH (A1253, Abclonal), anti-Transferrin (CSB-PA00250E0Rb, CUSABIO), anti-SLC40A1 (PHJ61901, AntibodySystem), anti-Pi3k (4292S, Cellsignaling), anti-Akt (WLP001a, Wanleibio), anti-Phospho Pi3k (17366, Cellsignaling), anti-Phospho Akt (WL0003b, Wanleibio), anti-BCL2 (CSB-RA564360A0HU, CUSABIO), anti-BAX (CSB-MA484077, CUSABIO). The membrane was then trimmed according to the size of the target protein. The membranes were then washed and probed with the appropriate horseradish peroxidase-conjugated secondary antibodies (As104, Abclonal) and detected using the ECL System.

### Statistical analysis

Data were analyzed and graphed using GraphPad Prism software, and all summary data are presented as mean ± s.e.m. Groups were compared using the student’s *t*-test. For the Kaplan-Meier survival plots, statistical significance was measured using the log-rank (Mantel-Cox) test. Compared with the control group, ^*^P < 0.05, ^**^P < 0.01, ^***^P < 0.001.

### Data availability

All study data are included in the Article and/or SI Appendix.

## Supplementary Materials

Supplementary Table 1
