# Supplementary material for: Capsaicin alleviates doxorubicin-induced acute myocardial injury by regulating iron homeostasis and PI3K-Akt signaling pathway
Source: Aging (Albany NY). 2023 Nov 1;15(21):11845–59. doi: 10.18632/aging.205138 (PMC10683596; doi:10.18632/aging.205138)
Supplement: Supplementary Table 1 [file aging-15-205138-s001.pdf]

SUPPLEMENTARY TABLE

Supplementary Table 1. The sequence of qPCR primer.

Name	Forward primer (5′–3′)	Reward primer (5′–3′)
BNP (mouse)	AAGTCCTAGCCAGTCTCCAGA	GAGCTGTCTCTGGGCCATTTC
GPX4 (mouse)	CGCGATGATTGGCGCT	CACACGAAACCCCTGTACTTATCC
Gapdh (mouse)	ATCATCCCTGCATCCACT	ATCCACGACGGACACATT
Myh7 (mouse)	GCTGAAAGCAGAAAGAGATTATC	TGGAGTTCTTCTCTTCTGGAG
Ptgs2 (mouse)	GCGACATACTCAAGCAGGAGCA	AGTGGTAACCGCTCAGGTGTTG
Gapdh (rat)	CCGCATCTTCTTGTGCAGTG	GAGAAGGCAGCCCTGGTAAC
Gpx4 (rat)	CCGGCTACAATGTCAGGTTT	ACGCAGCCGTTGTTATCAAT
Ptgs2 (rat)	ATGTTCGCATTCTTTGCCCAG	TACACCTCTCCACCGATGAC
